# Higher baseline global leukocyte DNA methylation is associated with MTX non-response in early RA patients

**DOI:** 10.1186/s13075-019-1936-5

**Published:** 2019-06-26

**Authors:** Helen R. Gosselt, Bertrand D. van Zelst, Maurits C. F. J. de Rotte, Johanna M. W. Hazes, Robert de Jonge, Sandra G. Heil

**Affiliations:** 1 Department of Clinical Chemistry, Erasmus MC, University Medical Center Rotterdam, P.O. Box 2040, 3000 CA Rotterdam, The Netherlands; 2 Department of Rheumatology, Erasmus MC, University Medical Center Rotterdam, Rotterdam, The Netherlands; 3 Department of Clinical Chemistry, Amsterdam Gastroenterology and Metabolism, Amsterdam UMC, Vrije Universiteit Amsterdam, Amsterdam, The Netherlands; 4 Department of Clinical Chemistry, Amsterdam Gastroenterology and Metabolism, Amsterdam UMC, Univ of Amsterdam, Amsterdam, The Netherlands; 5 Academic Center of Excellence - Inflammunity, Erasmus MC, University Medical Center Rotterdam, Rotterdam, The Netherlands

**Keywords:** DNA methylation, Arthritis, Tandem mass spectrometry, Methotrexate, Folic acid

## Abstract

**Background:**

Low-dose methotrexate (MTX) is the first-line therapy in early rheumatoid arthritis (eRA). Up to 40% of eRA patients do not benefit from MTX therapy. MTX has been shown to inhibit one-carbon metabolism, which is involved in the donation of methyl groups. In this study, we investigate baseline global DNA methylation and changes in DNA methylation during treatment in relation to clinical non-response after 3 months of MTX treatment.

**Methods:**

Two hundred ninety-four blood samples were collected from the Treatment in the Rotterdam Early Arthritis Cohort (tREACH, ISRCTN26791028), a multicenter, stratified single-blind clinical trial of eRA patients. Global DNA (hydroxy)methylation was quantified using liquid chromatography-electrospray ionization-tandem mass spectrometry (LC-ESI-MS/MS) and validated with a global DNA LINE-1 methylation technique. MTX response was determined as ΔDAS28. Additionally, patients were stratified into two response groups according to the European League Against Rheumatism (EULAR) response criteria. Associations between global DNA methylation and response were examined using univariate regression models adjusted for baseline DAS28, baseline erythrocyte folate levels, and body mass index (BMI).

**Results:**

Higher baseline global DNA methylation was associated with less decrease of DAS28 (*β* = 0.15, *p* = 0.013) and with MTX non-response (OR = 0.010, 95% CI = 0.001–0.188). This result was validated in LINE-1 elements (*β* = 0.22, *p* = 0.026). Changes in global DNA (hydroxy)methylation were not associated with MTX response over 3 months.

**Conclusions:**

These results show that higher baseline global DNA methylation in treatment naïve eRA patients is associated with decreased clinical response after 3 months of treatment of eRA patients and can be further evaluated as a predictor for MTX therapy non-response.

**Trial registration:**

ISRCTN, ISRCTN26791028, registered 23 August 2007—retrospectively registered

**Electronic supplementary material:**

The online version of this article (10.1186/s13075-019-1936-5) contains supplementary material, which is available to authorized users.

## Background

Rheumatoid arthritis (RA) is an autoimmune disease affecting about 1% of the world’s population [1]. The disease onset is unknown; nevertheless, medication can restrain disease activity and permanent joint damage. The disease-modifying anti-rheumatic drug (DMARD) methotrexate (MTX) is the first-line therapy in early rheumatoid arthritis (eRA) [2] and is often prescribed in combination with sulfasalazine (SSZ), hydroxychloroquine (HCQ), and corticosteroids. Up to 40% of treated patients do not adequately respond to therapy and need to switch to expensive biologicals after 3 to 6 months of therapy, or withdraw because of severe adverse events [3]. Therefore, new biomarkers are required to distinguish non-responders prior to treatment.

MTX is a folate antagonist of which the underlying mechanism in RA is still not fully elucidated. MTX was originally designed for cancer therapy to inhibit DNA synthesis by inhibiting key intracellular enzymes in folate metabolism. These include dihydrofolate reductase (DHFR) and thymidylate synthase (TS). The anti-inflammatory mechanism of action of low-dose MTX treatment used in eRA probably relates to the inhibition of key enzymes in the purine de novo synthesis pathway and release of anti-inflammatory adenosine [4]. MTX also inhibits methionine S-adenosyltransferase (MAT), followed by the inhibition of S-adenosyl methionine (SAM) in vivo and in vitro [5, 6]. SAM is responsible for the donation of methyl groups required for global DNA methylation. MTX is therefore hypothesized to inhibit global DNA methylation, although elevated global DNA methylation was observed in peripheral blood mononuclear cells (PBMCs) of MTX-treated patients [7]. If the effect of MTX is related to a decrease in global DNA methylation through inhibition of MAT and SAM, then we hypothesize that higher baseline global DNA methylation might be more difficult to inhibit and therefore affects MTX responsiveness.

In the current prospective study, we investigated whether higher baseline global DNA (hydroxy)methylation in leukocytes of early RA patients is associated with MTX clinical non-response over the first 3 months of treatment. Furthermore, we assessed whether a lesser decrease in global DNA methylation during treatment or higher global DNA methylation at 3 months of MTX treatment was associated with MTX clinical non-response.

## Materials and methods

### Subjects and samples

Four hundred ninety-six subjects were eligible from the Treatment in the Rotterdam Early Arthritis Cohort (tREACH, ISRCTN26791028), a multicenter, stratified single-blind, randomized controlled trial of eRA patients, as previously described [8]. In brief, included patients were diagnosed with RA based on the American College of Rheumatology (ACR) 1987 classification criteria for RA [9] and were categorized in high, intermediate, or low probability groups for persistent disease, according to the Visser prediction model [10]. All patients who received MTX mono or combination (MTX + corticosteroids and MTX + SSZ + HCQ + corticosteroids) therapy were enrolled in this study (*n* = 336). An escalating dose of MTX was prescribed in the first 3 weeks from 10 mg (week 1) up to 17.5 mg (week 2) and 25 mg (week 3). Additionally, all patients received weekly 10 mg folic acid, at least 24 h after MTX administration, as recommended [2]. Whole blood leukocytes were collected at baseline (T0) and after 3 months of MTX therapy (T3) and stored at − 80 °C. This study was approved by the medical ethics committee of the Erasmus University Medical Center: MEC-2006-252. Medical ethics committees at each participating center approved the study protocol, and written informed consent was obtained for all patients.

### LC-ESI-MS/MS

#### DNA digestion

Genomic DNA was isolated using the MagNA Pure Compact Nucleic Acid Isolation Kit (Roche Molecular Biochemicals®) according to the manufacturer’s instructions. DNA concentration was quantified using a NanoDrop ND-1000 Spectrophotometer with DNA-50 default settings (NanoDrop Technologies), and 260/280 ratios ~ 1.8 were considered pure DNA. Samples were stored at − 80 °C and diluted to 30 ng/μl 1 day prior to the start of the experiment. Six hundred nanograms of genomic DNA was added to the following digestion mixture: 1 μl DNA Degradase Plus™ enzyme (5 U/ml, Zymo Research®), 2.5 μl 10× DNA Degradase Reaction Buffer, and 1.5 μl Milli-Q, with a total reaction volume of 25 μl. The samples were centrifuged for 1 min at 3100 rpm and placed in a Thermo Mixer®C (Eppendorf) for 5 h at 37 °C, followed by an enzyme heat inactivation step for 20 min at 70 °C.

#### Quantification of global DNA (hydroxy)methylation

Following DNA degradation, the 25 μl reaction volume was 1:1 diluted with an Internal Standard mixture (IS, 19.2 nM 5-hmdc-d_3_, 205 nM 5-mdc-d_3_, 1.84 μM 2-dG-^15^N_5_). A calibration curve was made as follows: for each component, a calibrator was diluted to a final concentration of 10 nM (5-hmdC), 1000 nM (5-mdC), and 20,000 nM (2-dG), which were then serially 1:1 diluted to 0 in 5 steps. Of each dilution, 400 μl was added to 600 μl of diluted IS. Global DNA methylation and hydroxymethylation were measured using liquid chromatography-electrospray ionization-tandem mass spectrometry (LC-ESI-MS/MS) in the positive ionization mode. Twenty microliters was injected on a T3-high strength silica column (Acquity UPLC®, Waters, C18, 2.1 × 100 mm, 1.8 μm) at 35 °C. 0.1% formic acid in Milli-Q (A), and acetonitrile (B) was used as the mobile phase at a flow rate of 0.20 ml/min. The following gradient was used: 0–0.5 min (98% A and 2% B), 5 min (0% A and 100% B), 5.50 min (0% A and 100% B), 5.51 min (98% A and 2% B), and 7 min (98%A and 2%B), where all gradient steps were linear. An aliquoted DNA sample was measured as quality control (QC) in every run to uncover potential errors during sample preparation and DNA (hydroxy)methylation quantification. The coefficient of variation (%CV) was calculated from all the QC measurements (*n* = 24) and was 2.4% for methylation and 7.7% for hydroxymethylation measurements. The percentage of (hydroxy)methylation was calculated in relation to the total guanine concentration, using the following formulas:

$$ \%5\mathrm{mdC}=\left(\mathrm{nM}\ 5\mathrm{mdC}/\mathrm{nM}\ 2-\mathrm{dG}\right)\times 100 $$

$$ \%5\mathrm{hmdC}=\left(\mathrm{nM}\ 5\mathrm{hmdC}/\mathrm{nM}\ 2-\mathrm{dG}\right)\times 100 $$

### Sequenom EpiTYPER LINE-1 assay

LINE-1 global DNA methylation was determined in DNA from leukocytes, isolated using the Sequenom EpiTYPER® assay (Agena Bioscience™) as previously described [11, 12]. Briefly, 500 ng of purified genomic DNA was treated with sodium bisulfite to distinguish methylated from non-methylation cytosines using the EZ DNA MethylationTM Kit (Zymo Research®) according to the manufacturer’s instructions. Converted DNA concentrations were quantified using a ND-1000 NanoDrop Spectrophotometer (NanoDrop Technologies Inc.) using the RNA-40 default settings, as recommended (Zymo Research®). Bisulfite-converted DNA was stored no longer than 1 month at − 80 °C or until the experiment was performed. A LINE-1 bisulfite-targeted PCR was performed on the C-1000 Touch Thermal Cycler™ (Bio-Rad) using the following primers: 5′aggaagagagGTGTGAGGTGTTAGTGTGTTTTGTT-3′ and 3′cagtaatacgactcactatagggaggaaggctATATCCCACACCTAACTCAAAAAAT-′5. The PCR was followed by Shrimp Alkaline Phosphatase treatment, RNA transcription, and Sequenom analysis as previously described [12]. A mixture consisting of 100% enzymatically methylated DNA and 0% methylated DNA, due to a genetic knockout for methyltransferases, resulted in 50% DNA methylation and was used as a positive control during all steps. Milli-Q water was used as a negative control. DNA methylation was quantified using a Matrix-assisted Laser Desorption/Ionization-Time Of Flight (MALDI-TOF) MassARRAY® (Sequenom) analyzer according to the manufacturer’s instructions. Methylation percentage was calculated using the following formula: % Methylation = (area methylate peak)/(area unmethylated peak + area methylated peak) × 100. All samples were measured in triplet and samples with a variation coefficient (CV) of > 10% were checked for outliers by means of Dixon’s *Q* test. Outliers were removed, and if CV still exceeded 10% for the remaining duplicate, the sample was excluded. Twelve CpG sites were present within the LINE-1 PCR fragment of which CpG 6.7, CpG 8.9, and CpG 11.12 were combined, since these sites could not be separated. CpG 4 could not be analyzed because of a silent signal, and CpG 10 could not be analyzed due to a low mass fragment. Finally, the following seven CpG sites (CpG1, CpG2, CpG3, CpG5, CpG6.7, CpG8.9, and CpG11.12) were analyzed for differences in methylation [12].

### Statistical analysis

We performed paired analysis to assess the change in global DNA (hydroxy)methylation over the first 3 months using paired-sample *t* tests. Associations between global DNA (hydroxy)methylation at baseline, at 3 months and over 3 months (Δ(hydroxy)methylation) with response (ΔDAS28-ESR) were first analyzed in a univariate linear regression model, after which the associations were adjusted for confounders. Baseline DAS28 score, baseline erythrocyte folate levels, BMI, age, sex, and smoking status (current versus former + never) are known to be associated with MTX response and DNA methylation and were therefore tested as confounders [13–16]. The presence of anti-citrullinated protein antibodies (ACPA) has previously been related to decreased MTX response [17]. ACPA positivity was therefore tested as potential covariate. Confounders and covariates were only considered important when the effect size (beta coefficient, B) changed with > 10% upon adjustment. In addition, the relation between baseline global DNA methylation and MTX response was assessed dichotomously (non-responders versus moderate/good responders), according to the EUropean League Against Rheumatism (EULAR) response criteria at 3 months [18]. Associations between global DNA methylation and response were assessed in a crude logistic regression model and in a model adjusted for baseline DAS28, baseline erythrocyte folate, and BMI. Results are expressed in odds ratios (OR) with 95% confidence interval (CI). Incomplete cases were excluded prior to the analysis. For the correlation analysis, distributions of the variables were tested for normality using the Shapiro-Wilkinson test, where *p* > 0.05 was considered normally distributed. The correlation between baseline erythrocyte folate and global DNA methylation was tested using Spearman’s correlation due to the skewed distribution of erythrocyte folate, and the correlation between global DNA methylation determined by LC-ESI-MS/MS and LINE-1 was tested using Pearson’s correlation test (normally distributed variables). All statistical analyses were conducted using R Studio Software (Version 1.1.423; RStudio Team 2015), and *p* values< 0.05 were considered significant. Models tested for both methylation and hydroxymethylation were corrected for multiple comparisons using the Bonferroni correction, where *p* < 0.025 (0.05/2 = 0.025) was considered significant. LINE-1 analysis was corrected using the Bonferroni correction for the 7 CpGs that were tested simultaneously; hence, *p* < 0.007 (0.05/7 = 0.007) was considered significant.

## Results

### Subject baseline characteristics

Genomic DNA was available and isolated from leukocytes of 265 treatment-naive early RA patients and from 275 subjects at T3. A minimum of 600 ng was required for reliable (hydroxy)methylation measurements. Nine (T0) and five (T3) extracted DNA samples did not reach up to this minimum and were therefore excluded. Global DNA (hydroxy)methylation was successfully quantified in 294 patients, comprising 249 (T0) and 257 (T3) samples. Baseline characteristics of these 294 subjects are summarized in Table 1. The mean age was 53.4 ± 14.2 years, and 70.4% was female. Mean DAS28 at baseline was 4.7 ± 1.2 and decreased to 3.0 ± 1.2 over the first 3 months (Table 1). All patients were treatment naive at baseline and received MTX mono- or combination therapy for at least 3 months (Table 1).

**Table 1:** Baseline characteristics of early RA patients from the tREACH

	Mean ± SD
Patients, *N*	294
Male, *N* (%)	87.0 (29.6%)
Age (years)	53.4 ± 14.2
DAS28 score	4.7 ± 1.2
DAS28 score 3 months*	3.0 ± 1.2
Erythrocyte folate (nmol/L)*	936.0 ± 356.2
BMI (kg/m^2^)*	26.3 ± 5.1
Smoking status*	
Current, *N* (%)	91.0 (31.0%)
Never + former, *N* (%)	180.0 (61.2%)
ACPA status	
Positive, *N* (%)	193 (65.6%)
Treatment groups	
MTX, *N* (%)	54.0 (18.4%)
MTX + prednisone p.o., *N* (%)	81.0 (27.6%)
MTX + SSZ + HCQ + prednisone p.o., *N* (%)	83.0 (28.2%)
MTX + SSZ + HCQ + corticosteroids i.m., *N* (%)	76.0 (25.9%)

*Abbreviations*: *SD* standard deviation, *SSZ* sulfasalazine, *HCQ* hydroxychloroquine, *BMI* body mass index, *p.o*. per os, *i.m*. intramuscular

*Data was missing for DAS28 score at T3 (*n* = 10), baseline erythrocyte folate (*n* = 75), BMI (*n* = 3), and smoking status (*n* = 23)

### Global DNA hydroxymethylation increases during three months of MTX therapy

Mean global DNA methylation at baseline was 4.41 ± 0.13% and did not change significantly over the first 3 months of therapy (*p* = 0.454) (Additional file 1: Table S1). Global DNA hydroxymethylation increased significantly with 0.0008% over the first 3 months (*p* = 0.013; Additional file 1: Table S1).

### Higher baseline global DNA methylation is associated with MTX non-response at 3 months

Baseline global DNA methylation was associated with ΔDAS28 over the first 3 months when assessed in a crude univariate linear regression model (*B* = 1.36, *p* = 0.044). One percent difference in global DNA methylation at baseline corresponds to 1.41 difference in ΔDAS28 between patients, when adjusted for baseline DAS28, baseline erythrocyte folate, and BMI (*B* = 1.41, *p* = 0.013; Table 2).

**Table 2:** Associations between baseline global DNA (hydroxy)methylation and ΔDAS28 before and after 3 months of therapy

Methylation		Before MTX			After MTX		
		*B* (SE)	*β*	*p*	*B* (SE)	*β*	*p*
1	Methylation	1.36 (0.67)	0.15	0.044	0.40 (0.55)	0.05	0.471
2	Methylation	1.41 (0.56)	0.15	0.013	0.44 (0.47)	0.06	0.385
	DAS28	− 0.51 (0.06)	− 0.49	< 0.001	− 0.50 (0.06)	− 0.49	< 0.001
	Erythrocyte folate (nmol/L)	− 1.00 × 10^−3^ (2.00 × 10^−4^)	− 0.17	0.006	− 4.00 × 10^−4^ (2.00 × 10^−4^)	− 0.12	0.063
	BMI (kg/m^2^)	0.03 (0.02)	0.14	0.025	0.04 (0.02)	0.18	0.005
	Age (years)		–			–	
	Sex		–		0.27 (0.16)	0.11	0.098
	Smoking (current)		–		0.28 (0.16)	0.11	0.084
	ACPA status (positive)		–			–	
	Observations		181			179	
Hydroxymethylation		Before MTX			After MTX		
		*B* (SE)	*β*	*p*	*B* (SE)	*β*	*p*
1	Hydroxymethylation	19.56 (18.38)	0.08	0.288	12.52 (19.26)	0.05	0.517
2	Hydroxymethylation	6.90 (15.89)	0.03	0.664	5.92 (16.75)	0.02	0.724
	DAS28	− 0.54 (0.07)	− 0.52	< 0.001	− 0.52 (0.07)	− 0.51	< 0.001
	Erythrocyte folate (nmol/L)	− 1.00 × 10^−3^ (2.00 × 10^−4^)	− 0.18	0.007	−5.00 × 10^−4^ (2.00 × 10^−4^)	− 0.14	0.035
	BMI (kg/m^2^)	0.03 (0.02)	0.12	0.062	0.04 (0.02)	0.18	0.006
	Age (years)	0.01 (0.01)	0.10	0.125	0.01 (0.01)	0.13	0.057
	Sex	0.22 (0.17)	0.08	0.176	0.28 (0.16)	0.11	0.087
	Smoking (current)		–		0.28 (0.16)	0.11	0.090
	ACPA status (positive)		–			–	
	Observations		181			177	

Association between mean % global DNA (hydroxy)methylation and ΔDAS28 were tested in a crude univariate model (1) and adjusted for potential confounders (2). Potential confounders were baseline DAS28 score, baseline erythrocyte folate levels (nmol/L), BMI (kg/m^2^), age (years), sex, smoking status (current smoker versus former + never smoker), and ACPA status. Only biomarkers that changed the association with > 10% were considered confounders. *B* beta coefficient, *SE* standard error, *β* standardized beta coefficients. *p* < 0.05 was considered significant

In addition, we stratified subjects accordingly: non- and moderate/good responders according to the EULAR response criteria at 3 months.

**Fig. 1:**
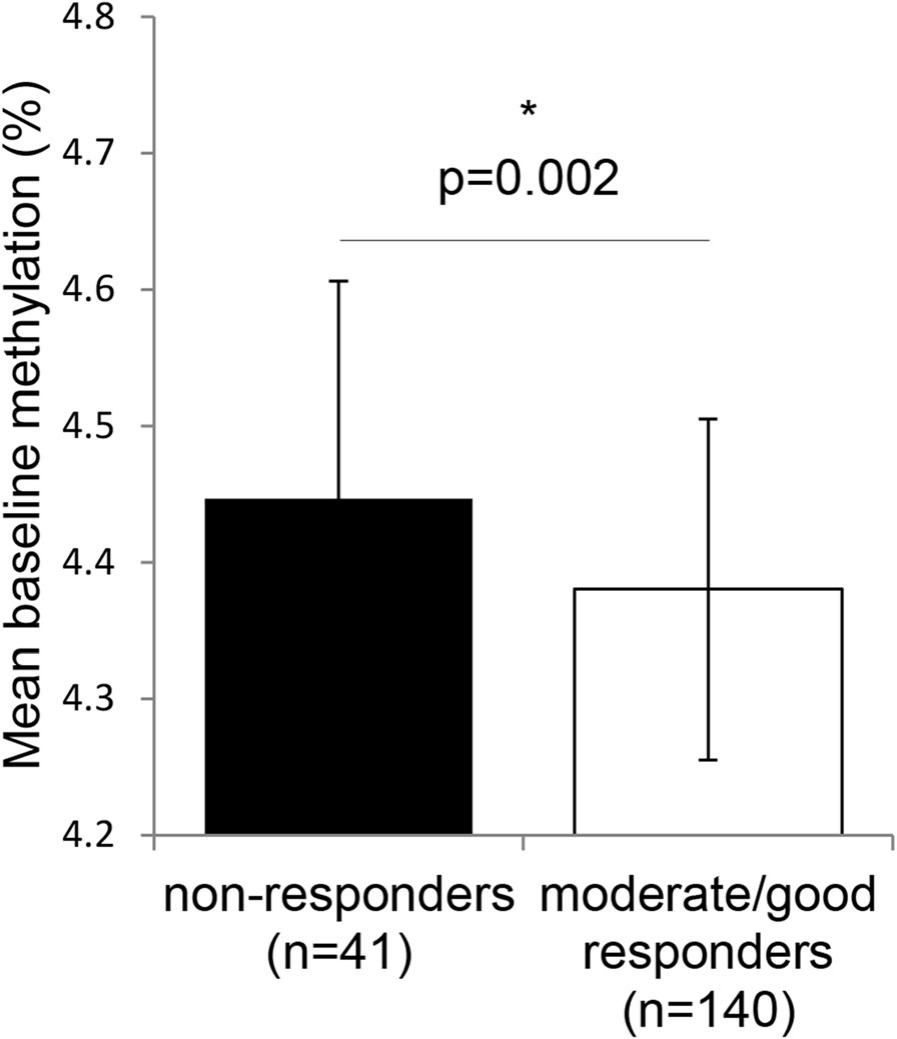
Higher mean (± SD) baseline global DNA methylation in EULAR non-responders compared to moderate/good responders. Response was determined according to the EULAR response criteria at 3 months. The *p* value is the result of a logistic regression analysis between baseline global DNA methylation and EULAR response criteria adjusted for baseline DAS28, baseline erythrocyte folate, and BMI. *P<0.05 was considered significant

Higher baseline global DNA methylation was associated with EULAR non-response in both a crude logistic model (OR = 0.027, 95% CI = 0.002–0.377) and when adjusted for baseline DAS28, baseline erythrocyte folate, and BMI (OR = 0.010, 95% CI = 0.001–0.188; Fig. 1).

Baseline global DNA hydroxymethylation was not significantly associated with ΔDAS28 in a crude univariate model (*B* = 19.56, *p* = 0.288), nor when adjusted for baseline DAS28, baseline erythrocyte folate, BMI, age, and sex (*B* = 6.90, *p* = 0.664; Table 2) and was therefore not further assessed between non-responders and moderate/good responders.

As folate is related to DNA methylation through one-carbon metabolism, we examined the correlation between baseline global DNA methylation and erythrocyte folate. We did not observe a correlation between baseline global DNA methylation and baseline erythrocyte folate concentrations (*R* = 0.084, *p* = 0.24).

### (Change in) global DNA methylation at 3 months is not associated with disease activity

Global DNA methylation at 3 months of therapy was not associated with ΔDAS28 (*B* = 0.40, *p* = 0.471), nor was global DNA hydroxymethylation at 3 months (*B* = 12.52, *p* = 0.517; Table 2). In addition, differences between DNA (hydroxy)methylation at baseline and after 3 months of therapy were not associated with changes in DAS28 (Δmethylation *B* = − 0.68, *p* = 0.182, Δhydroxymethylation *B* = − 1.55, *p* = 0.925; Table 3).

**Table 3:** Associations between changes in (hydroxy)methylation and in DAS28 over the first 3 months of therapy

		ΔMethylation			ΔHydroxymethylation		
	Biomarkers	*B* (SE)	*β*	*p*	*B* (SE)	*β*	*p*
1	Δ(hydroxy)methylation	− 0.50 (0.60)	− 0.07	0.403	− 9.32 (19.40)	− 0.04	0.632
2	Δ(hydroxy)methylation	− 0.68 (0.51)	− 0.09	0.182	− 1.55 (16.35)	− 0.01	0.925
	DAS28	− 0.51 (0.07)	− 0.51	< 0.001	− 0.52 (0.07)	− 0.51	< 0.001
	Erythrocyte folate (nmol/L)	− 1.00 × 10^−3^ (2.00 × 10^−4^)	− 0.15	0.027	− 1.00 × 10^−3^ (2.00 × 10^−4^)	− 0.16	0.024
	BMI (kg/m^2^)	0.05 (0.03)	0.19	0.005	0.04 (0.02)	0.18	0.008
	Age (years)	0.01 (0.01)	0.10	0.134	0.01 (0.01)	0.11	0.130
	Sex		–		0.20 (0.17)	0.08	0.240
	Smoking (current)	0.29 (0.17)	0.11	0.086	0.28 (0.17)	0.11	0.101
	ACPA status (positive)		–			–	
	Observations		163			161	

Associations were tested using crude univariate models (1) and adjusted for confounders (2). Potential confounders were baseline DAS28 score, baseline erythrocyte folate levels (nmol/L), BMI (kg/m^2^), age (years), sex, smoking status (current smoker versus former + never smoker), and ACPA status. Only biomarkers that changed the effect size with > 10% were considered confounders. *B* beta coefficient, *SE* standard error, *β* standardized beta coefficient. *p* < 0.05 was considered significant

### Higher LINE-1 methylation associated with decreased MTX response

LINE-1 global DNA methylation was determined in DNA isolated from leukocytes of 120 patients and was successfully quantified in 104 subjects. Seventy-eight individuals had no missing data in any of the variables needed in the analysis and were therefore used. LINE-1 methylation in CpG2 was not significantly associated with ΔDAS28 in a crude univariate model (*B* = 0.09, *p* = 0.242). However, it was associated with ΔDAS28 when adjusted for baseline DAS28, baseline erythrocyte folate, BMI, and smoking status (*B* = 0.16, *p* = 0.026; Table 4). Methylation at the other 6 CpG sites within LINE-1 was not associated with DAS28 nor was mean LINE-1 methylation (Additional file 1: Table S2).

**Table 4:** Validation of associations between global DNA methylation with ΔDAS28 (T3-T0) in LINE-1 CpG2

		Before MTX		
	Biomarkers	*B* (SE)	*β*	*p*
1	Methylation	0.09 (0.08)	0.13	0.242
2	Methylation	0.16 (0.07)	0.22	0.026
	DAS28	− 0.49 (0.09)	− 0.53	< 0.001
	Erythrocyte folate	− 1.00 × 10^−3^ (4.00 × 10^−4^)	− 0.12	0.197
	BMI	0.03 (0.02)	0.16	0.100
	Age	–	–	–
	Sex	–	–	–
	Smoking status	0.33 (0.23)	0.14	0.156
	ACPA status	–	–	–
	Observations		78	

Association between % baseline (T0) global DNA methylation in LINE-1 element CpG2 with ΔDAS28 (T3-T0), tested in a crude univariate model (1) and adjusted for potential confounders (2). Potential confounders were baseline DAS28 score, baseline erythrocyte folate levels (nmol/L), BMI (kg/m^2^), age (years), sex, smoking status (current smoker versus former + never smoker), and ACPA positivity. Only biomarkers that changed the association with > 10% were considered confounders. *B* beta coefficient, *SE* standard error, *β* standardized beta coefficient. *p* < 0.05 was considered significant

Furthermore, we examined the correlation between global DNA methylation obtained with LC-ESI-MS/MS and global DNA methylation in CpG2 assessed with the LINE-1 method. Here, we found a significantly positive correlation, although not strong (*R* = 0.34, *p* = 0.00061; Additional file 1: Fig. S1).

### Association baseline global DNA methylation and non-response strongest in MTX monotherapy group

To assess whether the association between baseline global DNA methylation and disease activity was specific for MTX response and not due to combination therapy, subjects were stratified by therapy and univariate linear regression analyses were performed. The effect size was 1.8-fold higher in the MTX monotherapy group (*B* = 2.06, *p* = 0.074) compared to the triple therapy group (*B* = 1.12, *p* = 0.173), although the associations were not significant (Table 5).

**Table 5:** Linear regression models for the association between global DNA methylation before MTX and ΔDAS28 over 3 months stratified by treatment group

Therapy	*N*	*B* (SE)	*β*	*p*
MTX	36	2.06 (1.12)	0.29	0.074
MTX + corticosteroids	48	1.51 (1.08)	0.18	0.172
MTX + SSZ + HCQ + corticosteroids	97	1.12 (0.81)	0.11	0.173

Associations were adjusted for baseline DAS28, baseline erythrocyte folate, and BMI. *MTX* methotrexate, *SSZ* sulfasalazine, *HCQ* hydroxychloroquine, *B* beta coefficient, *SE* standard error, *β* standardized beta coefficient. *p* < 0.05 was considered significant

## Discussion

In this study, we examined the association between global DNA (hydroxy)methylation, before-, at 3 months, and over 3 months of MTX therapy, in relation to changes in disease activity in leukocytes of eRA patients. We showed that higher baseline global DNA methylation is associated with clinical non-response, determined at 3 months of MTX treatment. This is in line with our hypothesis that higher baseline global DNA methylation levels are more difficult to inhibit and that this is associated with non-response. Furthermore, mean global DNA methylation did not change during MTX treatment, and global DNA methylation at and over 3 months was not associated with clinical efficacy. To our knowledge, we are the first to report an association between baseline global DNA methylation and early MTX response in eRA patients.

To predict response, associations prior to treatment are most suitable. Very few studies examined global DNA methylation status prior to treatment in relation to MTX response. Glossop and colleagues have identified 21 differentially methylated CpG sites in T-lymphocytes of 46 treatment-naive early RA patients. Of these, a combination of 1 hyper- and 1 hypomethylated CpG site gave the strongest predictive value [15]. A second study, in which 450k methylation arrays were performed, identified 2 baseline differentially methylated positions between 36 non-responders and 36 good responders that were associated with changes in c-reactive protein, but not with the complete DAS28 score [19].

Changes in global DNA methylation upon treatment were examined to give us more insight in the underlying mechanism. Despite the fact that MTX inhibits the universal methyl donor SAM, MTX administration has been shown to lead to increased global DNA methylation in peripheral blood mononuclear cells (PBMCs) of eRA patients [7, 20]. In contrast, we did not find significant methylation changes in leukocytes over the first 3 months. In our study, DNA was isolated from unsorted peripheral leukocytes. Leukocytes are a cell mixture of polymorphonuclear cells (PMN) and PBMCs, which have different methylomes. Changes in DNA methylation in PBMCs therefore might have been overshadowed, which possibly explains the different results between these and our study. In addition, in our study, all subjects were supplemented with folic acid, which stimulates methyl-group donation. In contrast to global DNA methylation, we did observe a small, but significant increase in global DNA hydroxymethylation during the first 3 months of therapy. Future studies are necessary to assess this observed effect of MTX on global DNA hydroxymethylation.

Previously, we demonstrated that lower baseline erythrocyte folate concentration was associated with non-response at 3 months [14]. Assuming that erythrocyte folate concentrations reflect folate concentrations in leukocytes, and knowing that folate donates one-carbon groups required for methylation reactions, a correlation with baseline DNA methylation was expected, despite we did not observe a correlation between baseline erythrocyte folate and baseline global DNA methylation. Furthermore, from the adjusted beta values in our model, we observed that baseline erythrocyte folate and global DNA methylation both explained ~ 15% of variation in DAS28, although the associations were in opposing directions. In addition, upon adjustment of the model for confounders, which included erythrocyte folate, we showed that the positive association between global DNA methylation and DAS28 is independent from baseline erythrocyte folate concentration.

According to the EULAR response criteria, response to therapy is determined at 6 months. However, the tREACH study is designed to produce the greatest treatment differences during the first 3 months of therapy [8], which is why we examined response over the first 3 months of therapy. Upon stratification by treatment, the association between baseline global DNA methylation and DAS28 was strongest in the MTX monotherapy group, despite the fact that this group was the smallest. This suggests that the association is regulated through MTX treatment. The associations upon stratification were not significant, which was probably due to a loss of power.

Strength of this study is that all patients received the same MTX dose due to the prospective study design of the tREACH. Moreover, DNA methylation and hydroxymethylation were quantified for each patient simultaneously with the same technique. Furthermore, the association between global DNA methylation and changes in disease activity upon MTX treatment were validated with a second technique. Limitations are that the majority of the patients received MTX-combination therapy and that the group size for LINE-1 methylation was limited, thus replication in larger MTX monotherapy studies is required. In addition, it would be interesting to examine DNA methylation in sorted peripheral blood leukocytes.

## Conclusions

In this paper, we showed that global DNA methylation is independently associated with disease activity over the first 3 months of MTX therapy. However, the underlying pathway, as well as the potential added value of global DNA methylation in a prediction model for MTX response requires further exploration.

## Additional file

Additional file 1: **Table S1.** Global DNA methylation and hydroxymethylation levels before MTX and three months after MTX therapy. **Table S2.** Linear regression models of %methylation in 6 LINE-1 CpG sites in relation to ΔDAS28 over three months of MTX therapy. **Figure S1.** Pearson correlation between global DNA methylation quantified using the LC-ESI-MS/MS and LINE-1 technique. (DOCX 153 kb)

## Data Availability

The dataset used and analyzed during the current study is available from the corresponding author on reasonable request.

## Abbreviations

%CV: Coefficient of variation
2-dG: 2′-Deoxyguanosine
5-hmdC: 2′-Deoxy-5-hydroxymethylcytidine
5-mdC: 2′-Deoxy-5-methylcytidine
ACR: American College of Rheumatology
DAS28-ESR: Disease activity score 28 based on the erythrocyte sedimentation rate
DHFR: Dihydrofolate reductase
DMARD: Disease-modifying anti-rheumatic drug
eRA: Early rheumatoid arthritis
EULAR: European League Against Rheumatism
HCQ: Hydroxychloroquine
IS: Internal standard
LC-ESI-MS/MS: Liquid chromatography-electrospray ionization-tandem mass spectrometry
MAT: Methionine S-adenosyltransferase
MTX: Methotrexate
PBMCs: Peripheral blood mononuclear cells
QC: Quality control
RA: Rheumatoid arthritis
SAM: S-Adenosyl methionine
SSZ: Sulfasalazine
tREACH: Treatment in the Rotterdam Early Arthritis Cohort
TS: Thymidylate synthase
