# Supplementary material for: Higher baseline global leukocyte DNA methylation is associated with MTX non-response in early RA patients
Source: Arthritis Res Ther. 2019 Jun 26;21:157. doi: 10.1186/s13075-019-1936-5 (PMC6595617; doi:10.1186/s13075-019-1936-5)

**Additional figures and tables**

| Additional Table 1. Global DNA methylation and hydroxymethylation levels before MTX and three months after MTX therapy | | | | |
| --- | --- | --- | --- | --- |
|  | N | Before MTX | After MTX | P |
| Methylation (%), mean ± SD | 212 | 4.41 ± 0.13 | 4.40 ± 0.16 | 0.454 |
| Hydroxymethylation (%), mean ± SD | 210 | 3.64x10^-2^ ± 5.00x10^-3^ | 3.72 x10^-2^ ± 5.00x10^-3^ | 0.013 |
| P-values are the result of a paired sample t-tests. P-values <0.05 were considered significant. | | | | |

| Additional Table 2 Linear regression models of %methylation in 6 LINE-1 CpG sites in relation to ΔDAS28 over three months of MTX therapy | | | | | | |
| --- | --- | --- | --- | --- | --- | --- |
|  | CpG1 | CpG3 | CpG5 | CpG6.7 | CpG8.9 | CpG11.12 |
| N | 79 | 78 | 78 | 79 | 79 | 79 |
| Mean% ±  SD | 66.77 ± 0.03 | 74.08 ± 1.81 | 38.34 ± 1.65 | 70.81 ± 2.26 | 70.39 ± 1.70 | 84.31 ± 1.37 |
|  | β (p) | β (p) | β (p) | β (p) | β (p) | β (p) |
| Methylation | -0.02 (0.816) | 0.09 (0.429) | 0.07 (0.474) | 0.13 (0.245) | 0.14 (0.269) | 0.13 (0.207) |
| DAS28 | -0.50 (<0.001) | -0.51 (<0.001) | -0.52 (<0.001) | -0.51 (<0.001) | - | -0.54 (<0.001) |
| Folate | -0.23 (0.021) | -0.22 (0.021) | -0.20 (0.034) | -0.22 (0.021) | -0.34 (0.003) | - |
| BMI | 0.12 (0.226) | 0.12 (0.208) | 0.12 (0.225) | 0.14 (0.148) | 0.21 (0.064) | 0.13 (0.178) |
| Age  Gender  Smoking | -  -  -  0.10 (0.330) | -  -  -0.01 (0.938)  0.11 (0.247) | -  -  -  - | -  -  0.01 (0.918) | -  -  -0.08 (0.521)  0.15 (0.170) | -0.05 (0.620)  -0.08 (0.391)  -  - |
| Potential confounders were: baseline DAS28 score, baseline erythrocyte – folate levels (nmol/L), BMI (kg/m^2^), age (years), gender and smoking status (current smoker versus former + never smoker). Only biomarkers that changed the association with >10% were considered confounders. β= standardized beta coefficient. P<0.05 were considered significant. | | | | | | |

Additional Figure 1. Pearson correlation between global DNA methylation quantified

and the LC-ESI-MS/MS and LINE-1 technique.


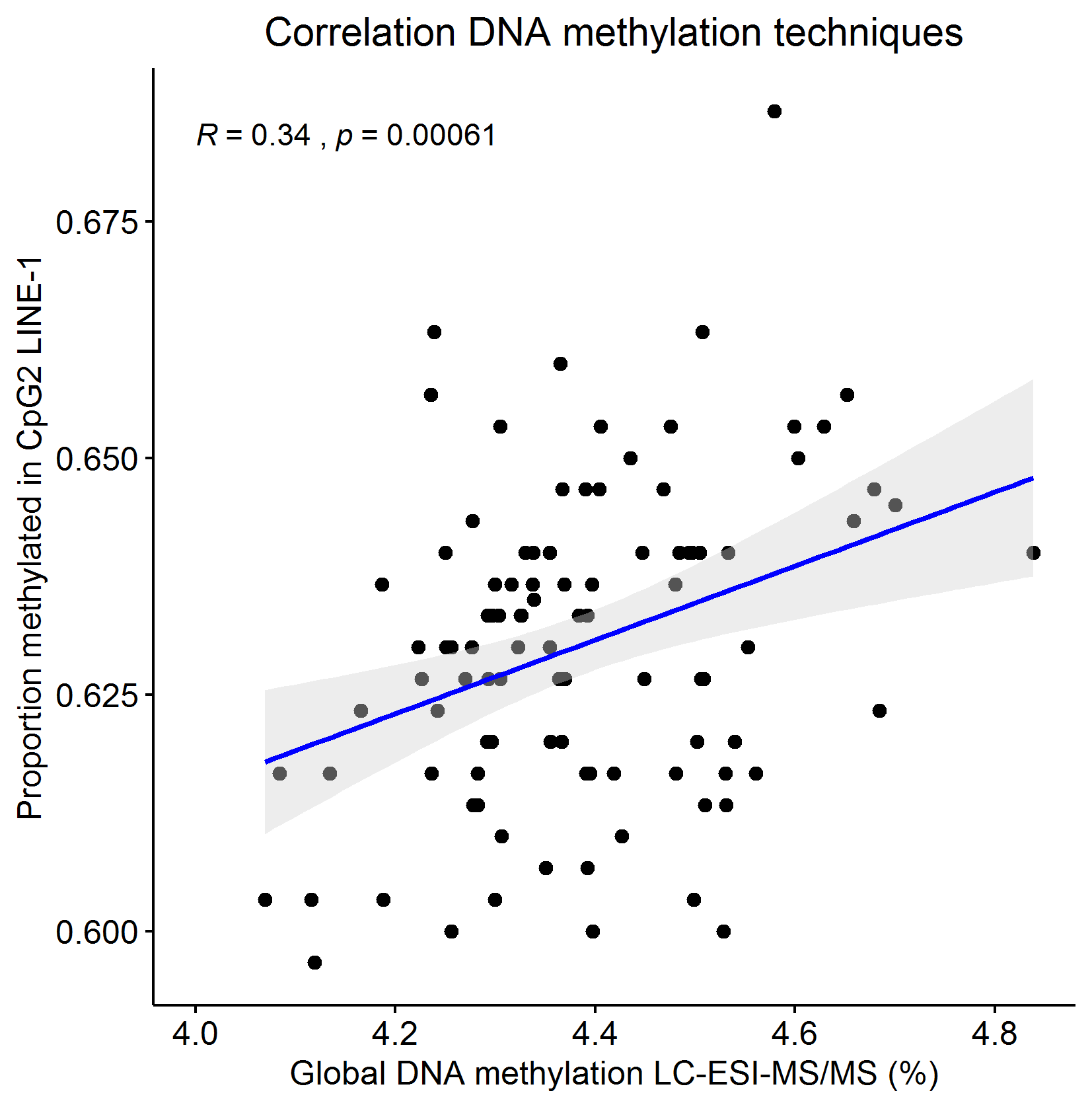

Supplement: Supplementary file 1 — Table S1. Global DNA methylation and hydroxymethylation levels before MTX and three months after MTX therapy. Table S2. Linear regression models of %methylation in 6 LINE-1 CpG sites in relation to ΔDAS28 over three months of MTX therapy. Figure S1. Pearson correlation between global DNA methylation quantified using the LC-ESI-MS/MS and LINE-1 technique. (DOCX 153 kb) [file 13075_2019_1936_MOESM1_ESM.docx]
